# Ebola Virus Antibody Prevalence in Dogs and Human Risk

**DOI:** 10.3201/eid1103.040981

**Published:** 2005-03

**Authors:** Loïs Allela, Olivier Bourry, Régis Pouillot, André Délicat, Philippe Yaba, Brice Kumulungui, Pierre Rouquet, Jean-Paul Gonzalez, Eric M. Leroy

**Affiliations:** * Centre International de Recherches Médicales de Franceville, Franceville, Gabon; † Centre Pasteur du Cameroun, Yaoundé, Cameroun; ‡ Institut de Recherche pour le Développement, Paris, France

**Keywords:** research, Ebola virus, dogs, antibody, human, prevention, reservoir

## Abstract

During the 2001–2002 outbreak in Gabon, we observed that several dogs were highly exposed to Ebola virus by eating infected dead animals. To examine whether these animals became infected with Ebola virus, we sampled 439 dogs and screened them by Ebola virus–specific immunoglobulin (Ig) G assay, antigen detection, and viral polymerase chain reaction amplification. Seven (8.9%) of 79 samples from the 2 main towns, 15 (15.2%) of 14 the 99 samples from Mekambo, and 40 (25.2%) of 159 samples from villages in the Ebola virus–epidemic area had detectable Ebola virus–IgG, compared to only 2 (2%) of 102 samples from France. Among dogs from villages with both infected animal carcasses and human cases, seroprevalence was 31.8%. A significant positive direct association existed between seroprevalence and the distances to the Ebola virus–epidemic area. This study suggests that dogs can be infected by Ebola virus and that the putative infection is asymptomatic.

## Abstract 2

This first report suggests that dogs can be asymptomatically infected with Ebola virus.

Ebola virus causes fulminant hemorrhagic fever in both humans and nonhuman primates (*1*,*2*). The Zaire Ebola virus species (Ebola virus-Z), 1 of the 4 known species of Ebola virus, occurs in central Africa and kills 80% of infected persons within a few days (*3*,*4*). Ebola hemorrhagic fever occurs in rare epidemics, in which the index patient is often infected by an animal source, which indicates that Ebola hemorrhagic fever is a zoonotic disease (*5*). During the past 3 years, 5 Ebola outbreaks due to Ebola virus-Z have struck the region of central Africa, including Gabon and Republic of Congo, and caused 334 deaths among the 428 reported human cases (*5*). In previous studies, we showed that each extended outbreak could be subdivided into several independent epidemic clusters or chains of transmission, which resulted from close contact with an Ebola virus-Z–infected animal carcass. Epidemiologic observations and genetic analyses identified gorilla, chimpanzee, and duiker carcasses as the main sources of human cases (*5*). Once the species barrier has been crossed between animals and humans, the disease spreads among humans by direct physical contact.

Some human cases in the recent outbreak in the Gabon/Republic of Congo region did not have a documented source of exposure to Ebola hemorrhagic fever. Similarly, 14 (4.9%) of the 284 cases in the 1976 Sudan outbreak (*6*) and 55 (17.4%) of the 316 cases during the 1995 outbreak in Kikwit (*7*), Democratic Republic of Congo (DRC, former Zaire), had no direct physical contact with an infected person or known infected carcass. These observations point to other routes of transmission (e.g., human-human respiratory tract infection through droplets and aerosols) or may suggest that other, unidentified animal sources may be involved in Ebola virus transmission to humans.

Ebola hemorrhagic fever outbreaks occurred in villages where people keep domestic animals, including dogs. The dogs are not fed and have to scavenge for their food. They eat small dead animals found near the villages and also internal organs of wild animals hunted and slaughtered by villagers. Some dogs are also used for hunting in the dense forested area. Although canine infection by Ebola virus has never been documented, domestic dogs’ behavior and diet place them at risk.

We examined whether pet dogs could have been infected by Ebola virus and their potential role as primary or secondary sources of human infection. We conducted a large-scale serologic survey to determine the prevalence of Ebola virus infection in pet dogs in an Ebola virus–epidemic area of Gabon.

## Methods

### Study Populations

**Figure 1:**
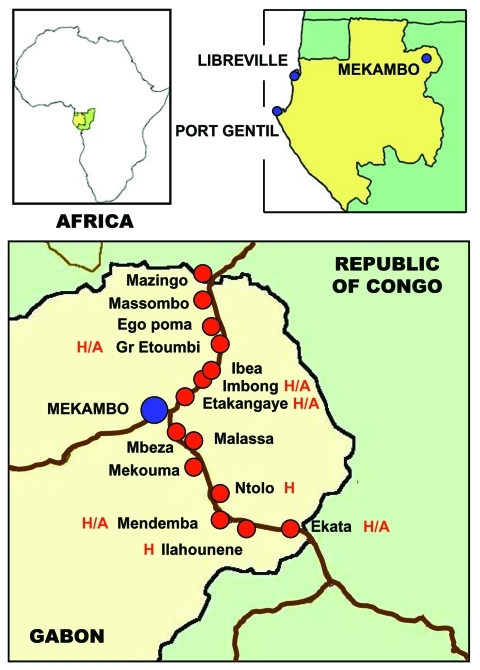
Locations of the main towns of Gabon (Libreville and Port Gentil) and the villages in the Ebola virus–epidemic area during the 2001–2002 outbreak in Gabon. The villages where human cases of Ebola infection were observed are indicated by “H.” The villages where both human patients and infected animal carcass were observed are indicated by “H/A.”

We sampled 439 dogs divided into 4 groups (Table 1). The first group comprised 102 dogs living in France (negative controls). The second group comprised 258 dogs sampled in the area of Gabon hit by the 2001–2002 Ebola outbreak. This group was subdivided into 2 clusters, 1 of 159 dogs from villages located between Mekambo and Ekata and between Mekambo and Mazingo (Figure 1, Table 1) and another of 99 dogs from Mekambo city, where human cases were also reported. The third group comprised 50 dogs from Libreville, the capital of Gabon, and 29 dogs from Port Gentil, Gabon’s second largest town, located on the Atlantic Coast (Figure 1, Table 1). Although these 2 Gabonese towns are both located >600 km from the Ebola virus–epidemic area, several human cases of Ebola infection, imported from the disease-epidemic area, were observed in Libreville during the 1996–1997 outbreak.

**Table 1:** Results of testing pet dogs for Ebola-specific immunoglobulin G antibodies by location

Location	No. dogs tested	No. dogs positive
Mekambo/Ekata		
Ekata	38	10
Ilahounene	15	1
Mendemba	3	1
Ntolo	11	3
Mekouma	12	1
Malassa	5	0
Mbeza	13	6
Total	97	22
Mekambo/Mazingo		
Mazingo	5	1
Massombo	1	0
Ego poma	4	1
Grand Etoumbi	7	3
Zoula	15	3
Ibea	12	3
Imbong	10	4
Etakangaye	8	3
Total	62	18
Mekambo	99	15
Libreville	50	5
Port Gentil	29	2
France	102	2
Total	439	64

### Sampling

Sampling was conducted in 2 ways. 1) Dogs in Libreville and Port Gentil were sampled in a veterinary clinic. Blood was collected in 5-mL dry Vacutainers (VWR International, Fontenay-sous-bois, France), and serum was prepared by centrifugation. Serum specimens were stored at –20°C until they were sent to the Centre International de Recherches Médicales de Franceville (CIRMF), Gabon, where they were stored at –80°C until testing. 2) Dogs from the Ebola virus–endemic area were sampled in the villages. An experienced veterinary team was located at Mekambo, where field laboratory facilities were set up; blood samples were collected on a daily basis in the vicinity of the village by using 5-mL dry Vacutainers and medetomidine anesthesia. The tubes were then transported to Mekambo each evening, and serum was decanted from whole blood. Serum samples were kept in liquid nitrogen in 1-mL aliquots at Mekambo until they were transported to CIRMF. Serum samples were then stored at –80°C until serologic testing, antigen detection, and RNA amplification were carried out.

Dog owners were interviewed on their pets’ activities (e.g., participation in hunting) and health history. The focus of the interviews was on potential Ebola virus–exposure events, including human cases that occurred in the village and among dog owners.

### Laboratory Investigations

Ebola virus–specific immunoglobulin (Ig) G was detected by using a standard enzyme-linked immunosorbent assay (ELISA) method as previously described (*8*). Briefly, Maxisorp plates (VWR International) were coated with Ebola virus–Z antigens diluted 1:1,000 in phosphate-buffered saline (PBS), overnight at 4°C. Control plates were coated with uninfected Vero cell culture antigens in the same conditions. Sera diluted 1:400 in 5% nonfat milk in PBS-Tween 20 (0.1%) were added to the wells and incubated overnight at 4°C. IgG binding was visualized by using a peroxidase-labeled anti-dog IgG (Kirkegaard & Perry Laboratories, Inc., Gaithersburg, MD, USA) and the TMB detector system (Dynex Technologies, Issy-les-Moulineaux, France). Optical density (OD) was measured at 450 nm with an ELISA plate reader. For each sample we calculated the corrected OD as the OD of the antigen-coated well minus the OD of the corresponding control well. The cut-off value (CO) was calculated as follows: CO = M + 3σ, where M is the average of the corrected OD of the 102 negative controls from France, and σ is the standard deviation. Samples were considered positive when the corrected OD was above the cut-off.

Samples positive in these serologic assays were used for antigen detection (*9*) and for viral polymerase chain reaction (PCR) amplification (*10*). Three positive and 3 negative serum specimens were also used for virus isolation (*9*). Briefly, Maxisorp plates were coated with a cocktail of 7 monoclonal antibodies against Ebola virus–Z antigens; control plates were coated with normal mouse ascitic fluid produced from the parent myeloma cell line. Serum was then added to the wells, followed by hyperimmune rabbit Ebola polyvalent antiserum and then peroxidase-conjugated goat antibodies against rabbit IgG. The TMB Microwell peroxidase substrate system was used to measure OD. For the detection of viral mRNA, total RNA was isolated from serum with the QIAmp viral RNA kit (Qiagen, Courtaboeuf, France), and cDNA was synthesized from mRNA as previously described. Two pairs of degenerate primers corresponding to the L-gene of Ebola virus were used for 2 rounds of amplification, which yielded a 298-bp fragment.

### Statistical Methods

Confidence intervals for proportions were calculated by using the Clopper and Pearson method (*11*). Statistical comparisons between seroprevalence rates according to the sampling area were performed by using the Fisher exact test. The Cochran-Armitage test was used as a trend test for proportions, after checking for the goodness-of-fit of the underlying linear model (*12*). All tests used a 0.05 significance level. Statistical analyses were performed by using R software (R Development Core Team; *13*).

## Results

A total of 439 blood samples from dogs were screened for Ebola virus–specific IgG. Two (2%) of the 102 blood samples from dogs living in France had detectable Ebola virus–reactive IgG (Table 2). Seven of the 79 dogs sampled in Libreville and Port Gentil (8.9% prevalence rate), 15 of the 99 dogs sampled in Mekambo (15.2% prevalence rate), and 40 of the 159 dogs sampled in villages located within the Ebola virus–epidemic area (25.2% prevalence rate) had detectable IgG to Ebola virus antigens (Table 2).

**Table 2:** Prevalence rates of Ebola-specific immunoglobulin G antibodies in pet dogs from different areas and villages

Area/village characteristic	No.	No. positive	Seroprevalence* (%)	95% confidence interval (%)
France	102	2	2	0.2–6.9
Major towns (Libreville and Port Gentil)	79	7	8.9	3.6–17.4
Mekambo	99	15	15.2	8.7–23.8
Ebola virus–epidemic area (villages)	159	40	25.2	18.6–32.6
Villages with human cases	92	25	27.2	18.4–37.4
Villages without human cases	67	15	22.4	13.1–34.2
Villages with human cases and animal source	66	21	31.8	20.9–44.4
Villages with human cases, without animal source	26	4	15.4	4.4–34.9
*Seroprevalence rates were compared by using the Fisher exact test with a 0.05 confidence level.				

During the 2001–2002 Ebola outbreak in Gabon, human cases of Ebola virus infection appeared only in certain villages within the Ebola virus–epidemic area (Figure 1). The prevalence of Ebola virus-reactive IgG among dogs from the villages where humans cases occurred was 27.2%, compared to 22.4% among dogs from villages where no human cases were noted (Table 2). In some cases, hunters had brought back to the village an Ebola virus–infected animal carcass found in the forest. This carcass was the source of human infection in the village, and the disease then spread from human to human, both within the village and to other villages by population movement (Figure 1). Thus, only secondary human cases were observed in some villages, with no identified animal source. The prevalence rate among dogs from villages with both an animal source and human cases was as high as 31.8%, compared to 15.4% among dogs from villages with human cases but no identified animal source (Table 2).

**Figure 2:**
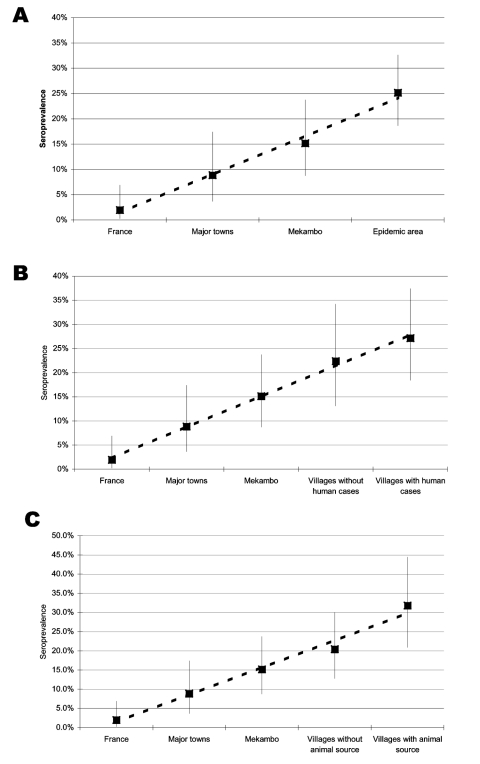
Seroprevalence of Ebola virus in dogs sampled in different areas: A) France, major towns of Gabon, Mekambo (a town close to the disease-epidemic area) and villages in the epidemic area; B) France, major towns of Gabon, Mekambo, villages without human cases and villages with human cases; C) France, major towns of Gabon, Mekambo, villages with and without an animal source. Estimates are represented by squares, bounded by their 95% Clopper and Pearson confidence intervals. The dashed line is the linear trend in proportion.

The seroprevalence rate was significantly lower in France (2.0%) than in Gabon (Table 2). In particular, it was lower than in the 2 major towns (p = 0.043), in Mekambo (p = 0.001), and in the Ebola virus-epidemic area (p < 0.001). The seroprevalence rate in the major towns (8.9%) was significantly lower than that in the Ebola virus–epidemic area (p = 0.003). Using scores from 1 to 4 for the canine prevalence rates in France, major towns, Mekambo and Ebola virus–epidemic areas, we observed a significant positive trend of linear increase (Cochran-Armitage test: p < 0.0001) (Figure 2A).

The seroprevalence rates in dogs increased linearly as the sampling area approached the sites of human cases, as confirmed by the highly significant Cochran-Armitage test for trends in proportions (p < 0.0001), which used a score of 1 for France, 2 for major towns, 3 for Mekambo, 4 for villages from the disease-epidemic area without human cases, and 5 for villages from the Ebola virus–epidemic area with human cases (Figure 2B). The result was confirmed when restricted to the 3 latter areas (p = 0.04).

In parallel, the seroprevalence rates in dogs increased linearly as the sampling area approached animal sources, as confirmed by a significant Cochran-Armitage test (p < 0.0001), using a score of 1 for France, 2 for major towns, 3 for Mekambo, 4 for villages where no animal source was observed (with or without human cases), and 5 for villages where an animal source was observed (with human cases) (Figure 2C). Again, the result was confirmed when restricted to the 3 latter areas (p = 0.01).

Neither Ebola virus antigens nor nucleotide sequences were detected in any of the positive or negative dog blood samples. We also failed to isolate the virus cells from 3 positive and 3 negative samples on Vero-E6.

## Discussion

We investigated the potential involvement of domestic dogs in the occurrence or dissemination of Ebola virus hemorrhagic fever in humans. Based on a large serologic survey of dogs in the 2001–2002 Ebola outbreak area in Gabon, we found evidence that dogs can be infected by Ebola virus, a finding that raises important human health issues. The ELISA method was based on the use of Ebola virus–Z antigens. Although cross-reactions can occur with antibodies to other subtypes, the presence of these subtypes in our samples is unlikely because only the Zaire subtype circulates in the study area: all patients and nonhuman primates tested in this part of central Africa were infected by the Zaire subtype alone. The 2 positive dogs in France, an apparently Ebola virus–exempt part of the world, could be attributed to false-positive reactions due to the calculation of the positivity cut-off and the 1:400 serum dilution step used in the tests.

We found that 40 of 159 dogs living in the 2001–2002 Ebola virus–epidemic area had detectable Ebola virus–specific IgG, indicating either true infection or simple antigenic stimulation. All the tests were standardized at the 1:400 serum dilution, and most serum specimens had high OD values even at higher dilutions, confirming the specificity of the reactions. These data are consistent with observations we made during the different Ebola outbreaks that occurred in Gabon and the Republic of Congo in recent years. We observed that some dogs ate fresh remains of Ebola virus–infected dead animals brought back to the villages, and that others licked vomit from Ebola virus–infected patients. Together, these findings strongly suggest that dogs can be infected by Ebola virus, and that some pet dogs living in affected villages were infected during the 2001–2002 human Ebola virus outbreak. No circulating Ebola antigens or viral DNA sequences (tested for by PCR) were detected in either positive or negative serum specimens, and attempts to isolate virus from these samples failed. These findings indicate either old, transient Ebola infection of the tested dogs, or antigenic stimulation.

Symptoms did not develop in any of these highly exposed animals during the outbreak, a finding that tends to support antigenic stimulation, asymptomatic, or very mild Ebola virus infection. Wild animals, especially gorillas and chimpanzees, can also be infected by Ebola virus, but the infection is highly lethal and causes huge outbreaks and massive population declines (*5*,*14*). Other animals such as guinea pigs (*15*), goats (*16*), and horses (*17*) remain asymptomatic or develop mild symptoms after experimental infection, but Ebola virus infection has never been observed in these species in the wild. Thus, dogs appear to be the first animal species shown to be naturally and asymptomatically infected by Ebola virus. Asymptomatic Ebola infection in humans has also been observed during outbreaks (*18*) but is very rare. Although dogs can be asymptomatically infected, they may excrete infectious viral particles in urine, feces, and saliva for a short period before virus clearance, as observed experimentally in other animals. Given the frequency of contact between humans and domestic dogs, canine Ebola infection must be considered as a potential risk factor for human infection and virus spread. Human infection could occur through licking, biting, or grooming. Asymptomatically infected dogs could be a potential source of human Ebola outbreaks and of virus spread during human outbreaks, which could explain some epidemiologically unrelated human cases. Dogs might also be a source of human Ebola outbreaks, such as the 1976 Yambuku outbreaks in Democratic Republic of Congo (*19*), the 1995 Kikwit outbreak, some outbreaks that occurred in 1996 and 2004 in Gabon and Republic of Congo (*5*), and the 1976 (*6*), 1979 (*20*), and 2004 (*21*) outbreaks in Sudan, the sources of which are still unknown. Together, these findings strongly suggest that dogs should be taken into consideration during the management of human Ebola outbreaks. To confirm the potential human risk of Ebola virus–infected dogs, the mechanisms of viral excretion (i.e. body fluids and virus kinetics of excretion) should be investigated during experimental canine infection. This research would also offer insights into the natural resistance of dogs.

The canine seroprevalence rates in Libreville and Port Gentil, the 2 main towns of Gabon, were significantly higher than that observed in France, which suggests antigenic stimulation in these towns where no cases of Ebola infection have been observed. Epidemiologic investigations showed that most seropositive dogs in Libreville and Port Gentil had probably never had contact with an infected source (dead animal or human case-patient), and that they had never visited the Ebola virus–epidemic area, in theory ruling out true infection. They may therefore have come into contact with free viral antigens, transmitted by aerosol or, to a lesser extent, experienced conjunctival exposure to virus-laden droplets of urine, feces, or blood of the unknown natural host. Ebola virus has been shown to be experimentally transmissible to rhesus monkeys by inhalation (*22*) and conjunctival exposure (*23*). Moreover, accidental transmission of Ebola virus to 2 rhesus monkeys that had no direct contact with experimentally infected monkeys was observed in a biocontainment laboratory, which also suggests aerosol, conjunctival, or oral transmission (*24*).

The Ebola virus reservoir species appears to extend throughout central Africa, both in rural and urban areas and might therefore be a small terrestrial mammal or a bird. No good candidate species has yet been identified, despite extensive studies (*25*,*26*). Epidemiologic observations during the 1976 outbreaks in Democratic Republic of Congo and Sudan identified bats as a potential reservoir (*6*,*20*), and Ebola virus nucleotide sequences and Ebola virus–like virus capsids were detected in rodents in the Central African Republic (*27*). The discovery of Ebola virus–positive pet dogs in undeclared affected areas suggests that these animals live in close contact with the Ebola virus reservoir, and this finding should help to narrow the search.

One striking result of this study is the significant increasing gradient of canine seroprevalence from France to the Ebola virus–epidemic area, including from villages with and without human cases in the area. The Cochran-Armitage test for trends in proportions showed that seroprevalence increased linearly from France (2%), to major towns (8.9%), then to Mekambo (15.2%), and then to villages in the Ebola virus–epidemic area (25.2%). This trend is supported by the increasing seroprevalence as the sampling area approached human cases and animal sources (Cochran-Armitage test, p < 0.0001). These findings suggest that canine seroprevalence could reflect contact with the virus and, thus, virus activity in a given area and also the risk for human infection.

The virus appears to jump from its natural host to humans only in specific, but unknown, conditions. Seroprevalence rates in dogs might serves as an indicator of Ebola virus in regions in which no animal deaths or human cases have been observed.

In conclusion, this study offers the first evidence that dogs might be asymptomatically infected by Ebola virus in the wild. This finding has potential implications for preventing and controlling human outbreaks. The increasing canine seroprevalence gradient from low-risk to at-risk Ebola virus–endemic areas indicates that this seroprevalence might be used as an epidemiologic indicator of virus circulation in regions where no other means of virus detection are available.
